# Supplementary material for: Rosetta design with co-evolutionary information retains protein function
Source: PLoS Comput Biol. 2021 Jan 19;17(1):e1008568. doi: 10.1371/journal.pcbi.1008568 (PMC7815116; doi:10.1371/journal.pcbi.1008568)
Supplement: S1 Supplement — (PDF) [file pcbi.1008568.s001.pdf]

Section 1

Benchmak Protein Description

Here, additional data on the other benchmark proteins can be found, highlighting the broad use of ResCue. For a detailed description of the protein design methods used, see Section 2.

1.1 Overview of all ten benchmark proteins

In all cases ResCue designs showed a lower energy increase compared to SeqProf and RECON, while having a large increase in crs values (Fig S1). All ResCue designs occupied a more favorable area of this energy landscapes. In all of our benchmark proteins there is a clear separation of the design methods visible.

1.2 A network of coupled residues is involved in the binding of ATP in the HPPK

The 6-Hydroxymethyl-7,8-dihydropterin pyrophosphokinase (HPPK) belongs to a class of enzymes catalyzing the pyrophosphoryl transfer from ATP to 6-hydroxymethyl-7,8-dihydropterin (HP), which is the first reaction in the folate pathway (1; 2; 3). Here, the residue interaction network formed by the 20% highest coupled residues $res_{cc}^{20}(HPPK)$ is mostly involved in the binding of ATP (Q74, R88, W89, H115, Y116, R121) (Fig S2, red network). In contrast, the other found small networks (purple, green, yellow) are not annotated in the literature. Other residues critical for the substrate/cofactor binding besides ATP were not part of our found networks, but some of the residues are conserved. This conservation excludes co-evolutionary mutations with other residues. Comparing the sequence logos of designed sequences reveals that there is almost no difference in sampling between RoSSD and RECON MSD (Fig S3). This lack of improvement is not surprising since the RMSD between the two conformational states is only 0.5Å, and optimizing over two almost identical state has no additional benefit. In the case of the two residues H115 and R121, sequences designed with SeqProf constraints sample the native amino acid more often than sequences designed with ResCue. Analyzing the MSA revealed that these two positions are conserved residues, preventing a co-evolutionary signal.

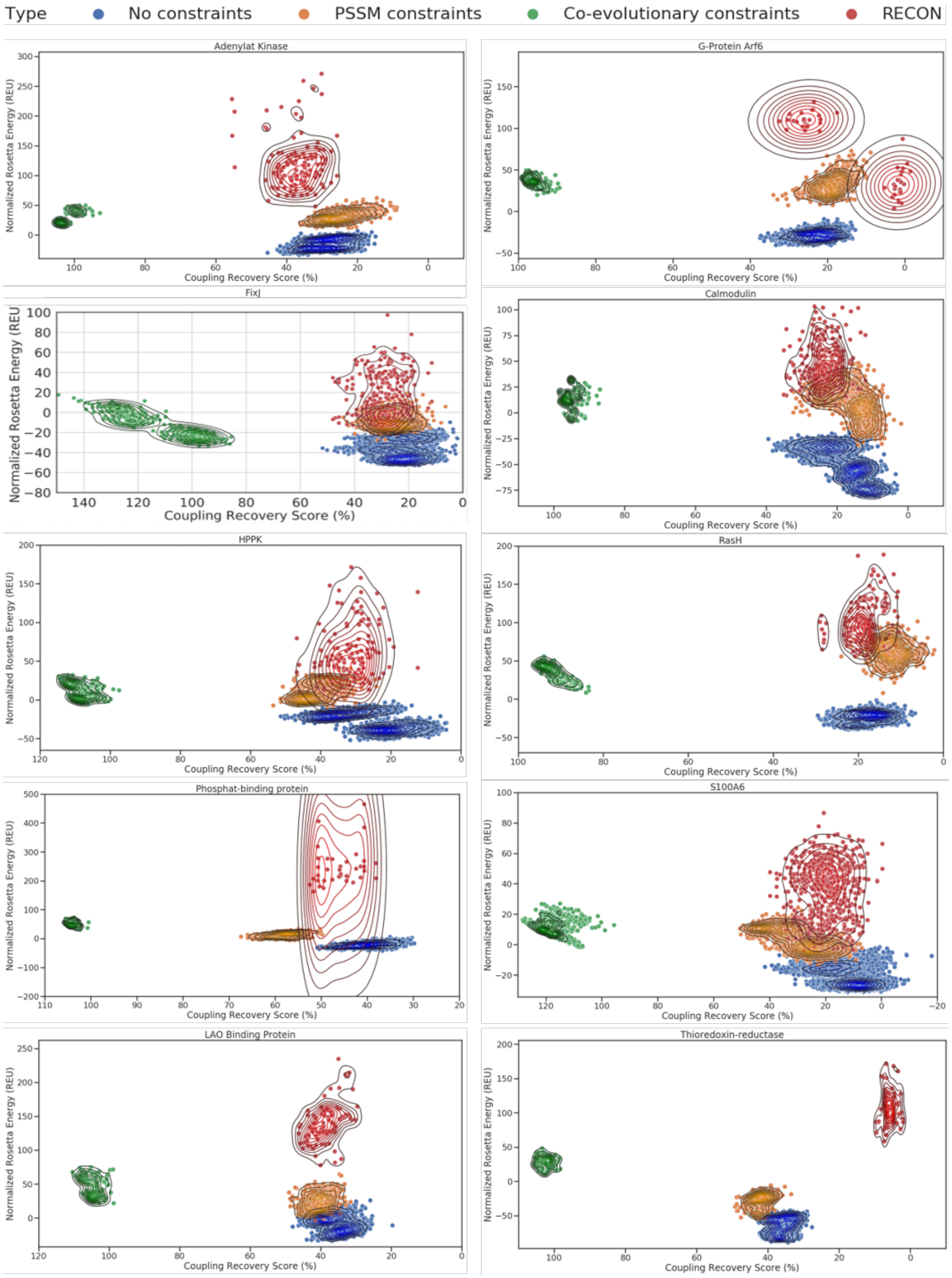

Fig S1. Energy landscapes for designed sequences. The normalized Rosetta Energy in REU is plotted against the Coupling Recovery Score crs (%). Each dot represents a designed sequence and is colored by the used design protocol. Each plot is one of the ten proteins used as benchmark. (Note that in typical Rosetta fashion the x-axis is inverted, to highlight the energy funnel. Additionally the crs is represented in % to enable comparison between the proteins.)

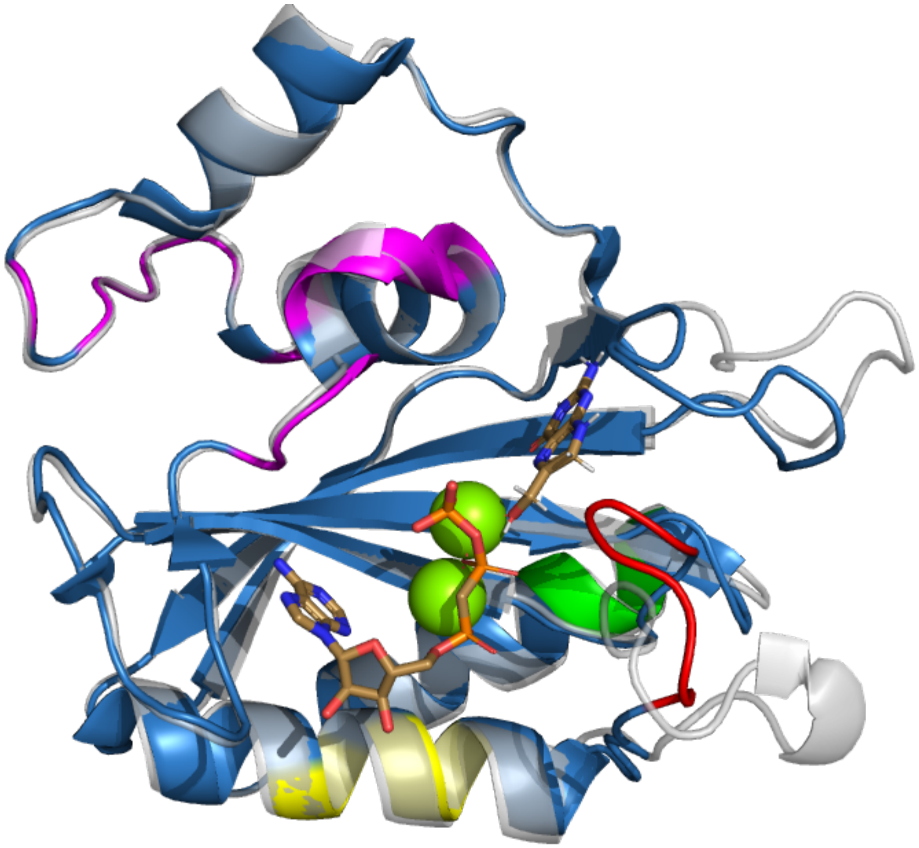

Fig S2. Localization of highly coupled residues in HPPK. Network of highly coupled residues (red, yellow, green, purple) displayed on the structure of HPPK (PDB ID: Unbound 1HKA, Bound 1Q0N). Alignment of the bound state (blue) and the unbound state (grey). The substrate ATP and a HP analog are shown as sticks. Bound magnesium is depicted as green spheres.

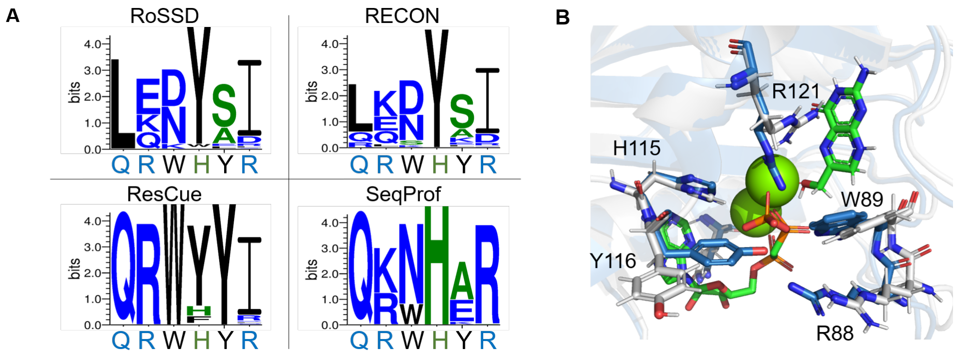

Fig S3. Sequence logos resulting from four design protocols for HPPK. The native sequences are listed below the logos. (**A**) Shown are sequence logos for six binding site residues for each design approach. (**B**) Graphical view of the binding site residues and the substrates ATP and a HP analog (green) shown in stick representation. Native structure in closed state is depicted in blue, while a protein designed with ResCue is shown in grey. For the protein designs, the ligands were not part of the starting structure. (Bound state PDB ID: 1Q0N)

1.3 The calcium sensor mechanism of S100A6 relies on the coupled residues at the two binding sites

S100A6 is a member of the S100 family of calcium-binding proteins and undergoes a conformational shift after binding (4). The protein functions as a calcium sensor through a helix-loop-helix motif called EF-hand, similar to calmodulin and troponin C (5). Here, the residue interaction network formed by the 20% highest coupled residues $res_{cc}^{20}(S100A6)$ is separated into two distinct networks at the two calcium-binding sites (Fig S4). Interestingly, while the red network at the second binding site captures all crucial binding residues, the yellow network at the first binding site is small and only in close spacial proximity. This result is perhaps explained by the fact that the coordination of the calcium in the first site primarily involves main chain carbonyls compared to the involvement of side chains in the second binding site.

Comparing the sequence logos of residues crucial for calcium-binding at both sites of different design approaches further demonstrates the difference between the two binding sites (Fig S5). Here, we expected RECON do perform better at sampling the native amino acids at the second binding site than the first. This expectation is based on the fact that a large conformational shift occurs at the second binding site while the first site shows almost no change. Indeed, RECON samples the native amino acids at the second binding site more often than at the first. While the residues at the first binding site are already well sampled by RoSSD design, the residues at the second site have only in ResCue designs the native amino acids.

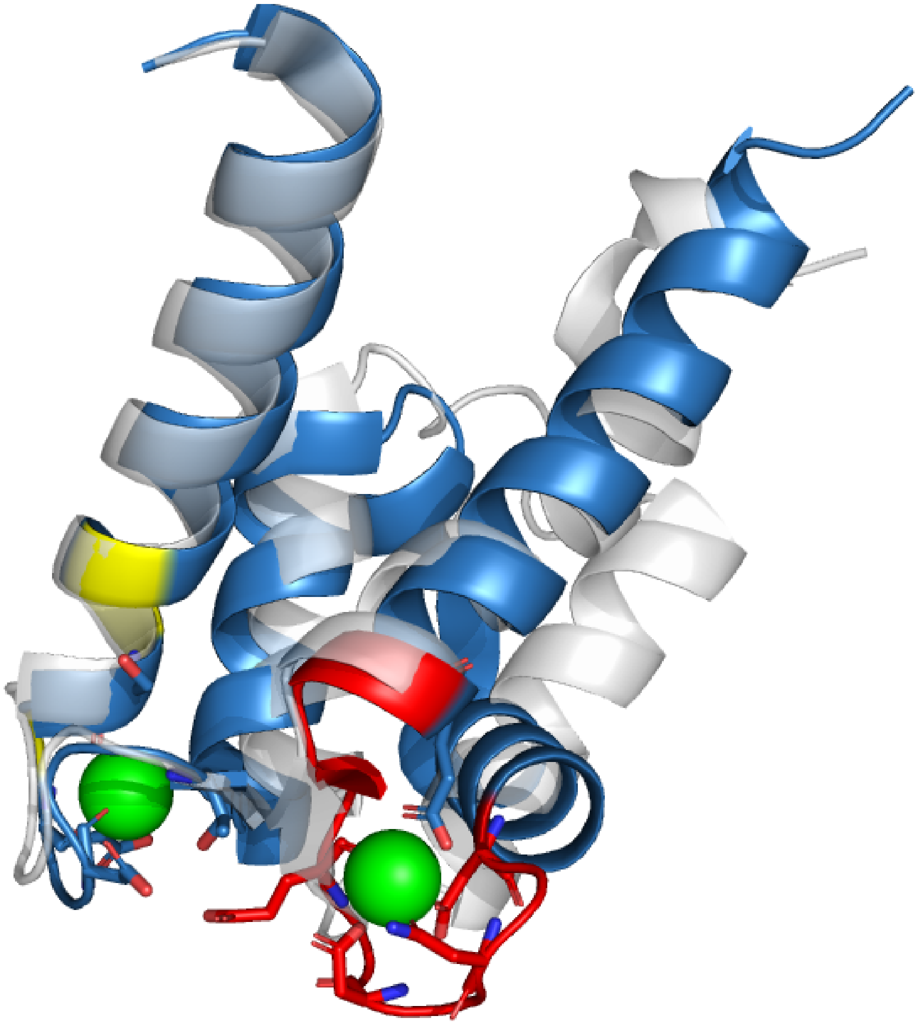

Fig S4. Localization of highly coupled residues in S100A6. Network of highly coupled residues (red, yellow) displayed on the structure of S100A6 (PDB ID: Calcium bound 1K9K, Calcium free 1K9P). Alignment of the calcium bound state (blue) and the calcim free state (grey). Bound magnesium is depicted as green spheres. Residues critical for calcium-binding are shown as sticks.

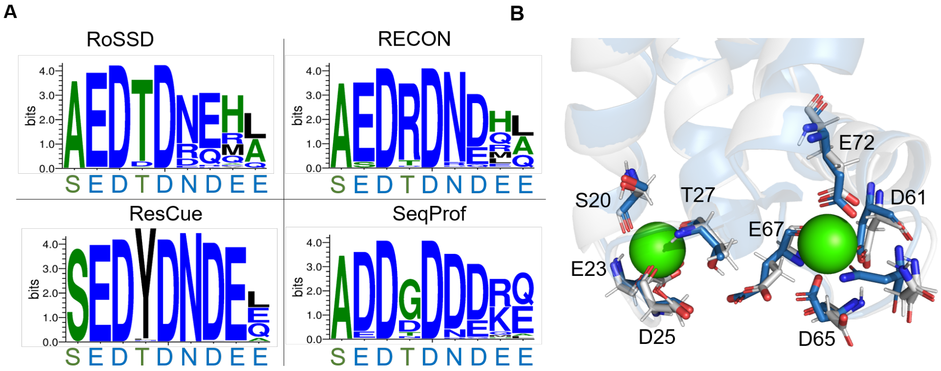

Fig S5. Sequence logos resulting from four design protocols for S100A6. The native sequences are listed below the logos. (**A**) Shown are sequence logos for nine binding site residues for each design approach. (**B**) Native structure in calcium bound state is depicted in blue, while a protein designed with ResCue is shown in grey. For the protein designs, the ligands were not part of the starting structure. (Bound state PDB ID: 1K9K)

1.4 A network of coupled residues contributes to the conformational shift after phosphate binding in the Phosphate-Binding Protein

The phosphate-binding protein (PBP) is responsible for the active transport of phosphate in bacterial cells and is highly specific for phosphate. The binding of phosphate is stabilized by twelve hydrogen bonds, as well as one salt link (6). We found that the residue interaction network formed by the 20% highest coupled residues $res_{cc}^{20}(PBP)$ connects the positions forming hydrogen bonds with the phosphate with residues further away from the binding site that undergo conformational changes upon binding (Fig S6, Residues T10, F11, A13, Y33, S38, D56, N137,R135, S139, G140, T141, S142, G176, N177, E195, Y198, T256, F257). Analyzing the designed sequences of our different approaches with sequence logos highlighted how only our ResCue approach samples the residues necessary to create hydrogen bonds with the ligand (Fig S7). The only residue that is not sampled in all different methods is R135, which forms a hydrogen bond with a water molecule. Again, water is not commonly included in protein design and thus remains a challenge for Rosetta.

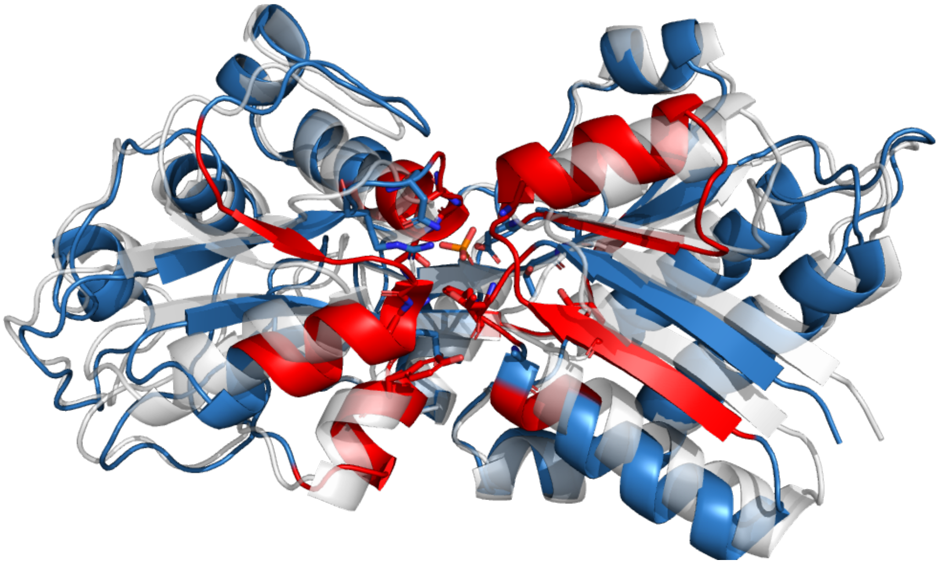

Fig S6. Localization of highly coupled residues in PBP. Network of highly coupled residues (red) displayed on the structure of PBP (PDB ID: Unbound 1OIB, Bound 1QUK). Alignment of the bound state (blue) and the unbound state (grey). The substrate phosphate is shown as sticks. Residues known to be crucial for substrate binding are shown as sticks.

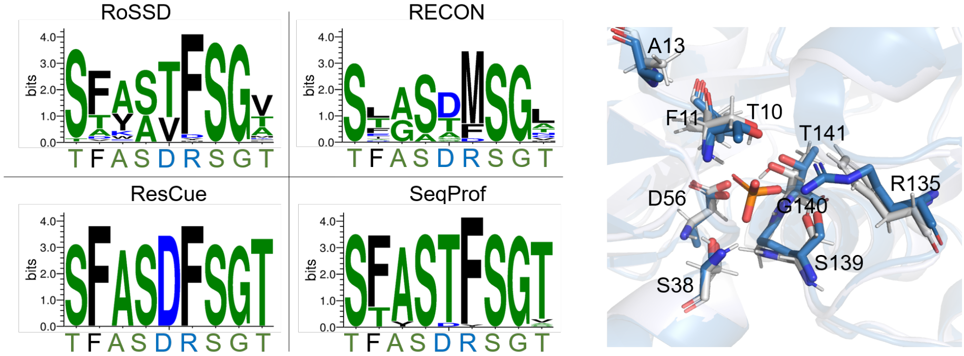

Fig S7. Sequence logos resulting from four design protocols for PBP. The native sequences are listed below the logos. (**A**) Shown are sequence logos for nine binding site residues for each design approach. (**B**)Graphical view of the binding site residues and the ligand phosphate (orange) shown in stick representation. Native structure in closed state is depicted in blue, while a protein designed with ResCue is shown in grey. For the protein designs, the ligand was not part of the starting structure. (Bound state PDB ID: 1QUK)

1.5 A network of coupled residues is involved in the binding of GTP in the small G protein Arf6-GDP

Arf6 localizes at the periphery of the cell and plays an essential role in endocytotic pathways (7; 8). Here, the residue interaction network formed by the 20% highest

coupled residues $res_{cc}^{20}(Arf6)$ is involved in the binding of GTP (T41, I42, D63, V64, G65, G66) (Fig S9, red network). Comparing the sequence logos of designed sequences reveals that RoSSD often samples the native amino acids at positions 41, 63, 64 and 65. RECON does a slightly better job at sampling the native sequence, for example in the case of G66 (Fig S8). In the case of the residues I42 and D63 and G66, only ResCue designs show a clear bias towards the native sequence.

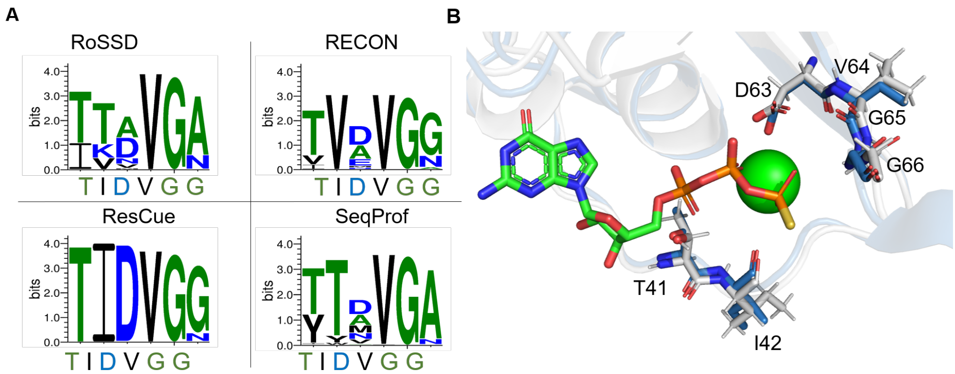

Fig S8. Sequence logos resulting from four design protocols for Arf6. The native sequences are listed below the logos. (**A**) Shown are sequence logos for six binding site residues for each design approach. (**B**) Graphical view of the binding site residues and the ligand shown in stick representation. Native structure in GTP bound state is depicted in blue, while a protein designed with ResCue is shown in grey. For the protein designs, the ligand was not part of the starting structure. (PDB ID: With GDP 1E0S, With GTP 2J5X)

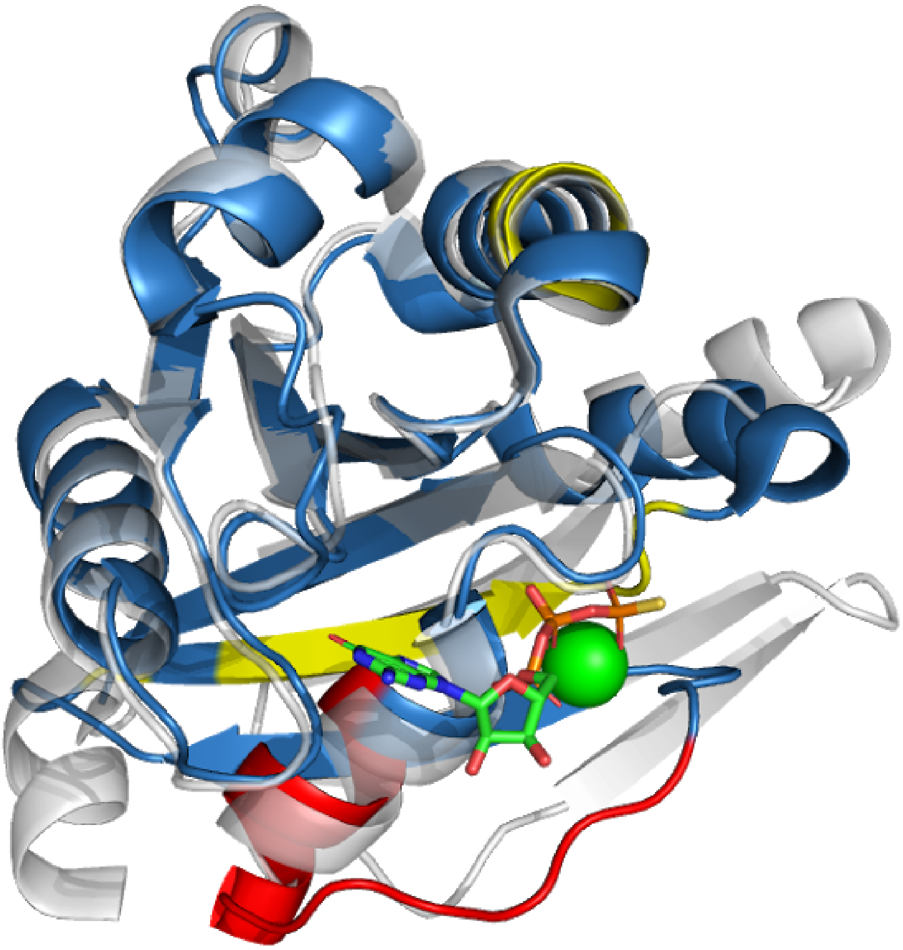

Fig S9. Localization of highly coupled residues in Arf6. Network of highly coupled residues (red) displayed on the structure of Arf6 (PDB ID: With GDP 1E0S, With GTP 2J5X). Alignment of the GTP bound state (blue) and the GDP bound state (grey). The substrate is shown as sticks.

1.6 A network of coupled residues is involved in the binding of AMP in the Adenylate Kinase

Adenylate kinases are nucleoside monophosphate (NMP) kinases and consist of a large CORE domain, a small NMP-binding domain and a LID domain (9; 10). Here, the residue interaction network formed by the 20% highest coupled residues $res_{cc}^{20}(AdenylatKinase)$ is involved in the binding of AMP (Network residues: 29-40, 57, 58, 60, 61, 81-93, Binding residues: T31, R36, K57, L58, G85, F86, P87, R88) (Fig S10, red network). Comparing the sequence logos of designed sequences reveals that all methods sample the native amino acids for G85, F86 and P87 (Fig S11). However, in the case of the residues T31, R36 and K57, only ResCue designs show a clear bias towards the native sequence.

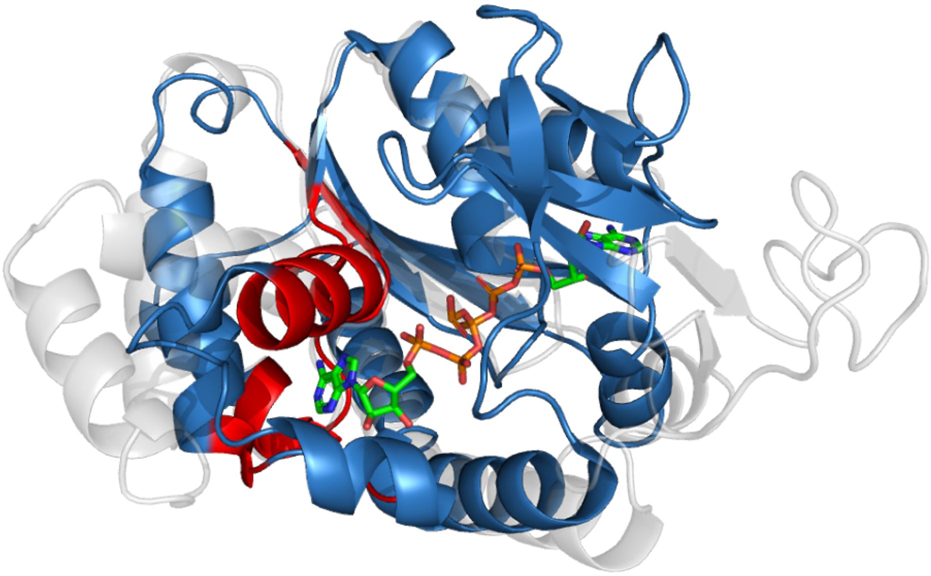

Fig S10. Localization of highly coupled residues in the Adenylate kinase. Network of highly coupled residues (red) displayed on the structure of the Adenylate kinase (PDB ID: Ap5A bound 1AKE, unbound 4AKE). Alignment of the Ap5A bound state (blue) and the unbound state (grey). The substrate is shown as sticks.

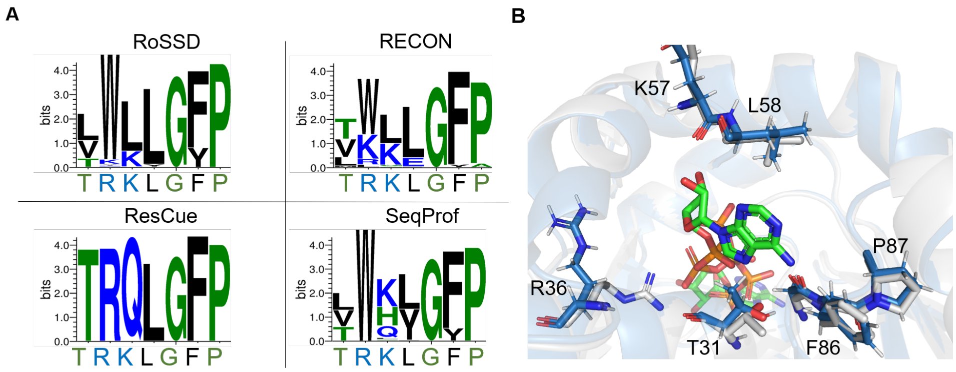

Fig S11. Sequence logos resulting from four design protocols for the Adenylate kinase. The native sequences are listed below the logos. (**A**) Shown are sequence logos for seven binding site residues for each design approach. (**B**) Graphical view of the binding site residues and the ligand shown in stick representation. Native structure in Ap5A bound state is depicted in blue, while a protein designed with ResCue is shown in grey. For the protein designs, the ligand was not part of the starting structure. (PDB ID: Ap5A bound 1AKE, unbound 4AKE)

1.7 A network of coupled residues is involved in the binding of FAD in the Thioredoxin reductase

In the thioredoxin reductase, cycles of reduction and reoxidation of FAD depend on rate-limiting rearrangements of the FAD and NADPH domains (11; 12). Here, the

residue interaction network formed by the 20% highest coupled residues $res_{cc}^{20}(Thioredoxinreductase)$ is involved in the intearction with FAD (Network residues: 44-53, 132-144, 159-168, 172, 173, 179-182, 184, 291-309, Binding residues: N51, R181, R293, Q294, A295) (Fig S12, red network). Comparing the sequence logos of designed sequences reveals that RoSSD samples the native amino acids for N51 and Q294(Fig S13). However, in the case of the residues R181 and R293, only ResCue designs show a clear bias towards the native sequence.

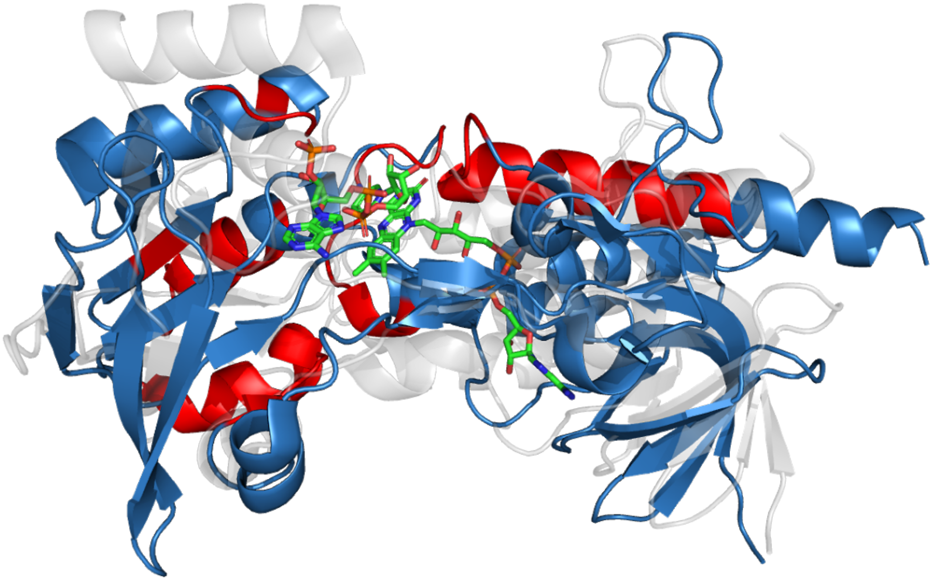

Fig S12. Localization of highly coupled residues in the Thioredoxin reductase. Network of highly coupled residues (red) displayed on the structure of the Thioredoxin reductase (PDB ID: AADP+ bound 1F6M, unbound 1E0S). Alignment of the AADP+ bound state (blue) and the unbound state (grey). The substrate is shown as sticks.

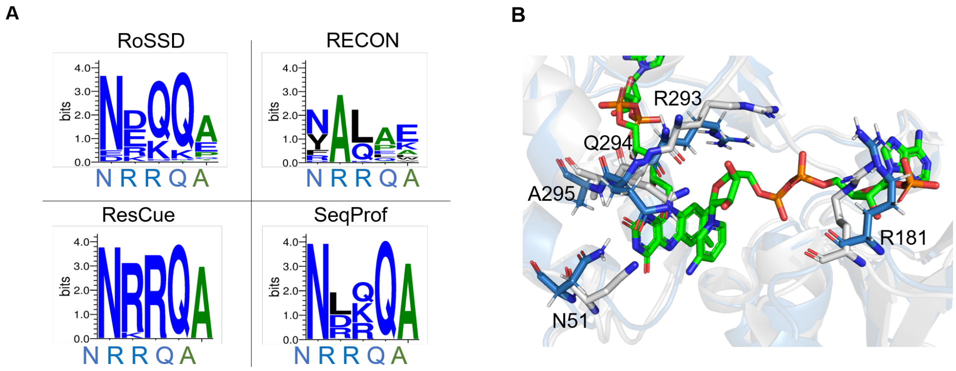

Fig S13. Sequence logos resulting from four design protocols for the Thioredoxin reductase. The native sequences are listed below the logos. (**A**) Shown are sequence logos for five binding site residues for each design approach. (**B**) Graphical view of the binding site residues and the ligand shown in stick representation. Native structure in AADP+ bound state is depicted in blue, while a protein designed with ResCue is shown in grey. For the protein designs, the ligand was not part of the starting structure. (PDB ID: AADP+ bound 1F6M, unbound 1E0S)

Bibliography

1. Xiao B, Shi G, Chen X, Yan H, Ji X. Crystal structure of 6-hydroxymethyl-7, 8-dihydropterin pyrophosphokinase, a potential target for the development of novel antimicrobial agents. Structure. 1999;7(5):489–496.

2. Switzer RL. In: 19. Phosphoribosylpyrophosphate Synthetase and Related Pyrophosphokinases. vol. 10. Elsevier; 1974. p. 607–629.

3. Blaszczyk J, Shi G, Yan H, Ji X. Catalytic center assembly of HPPK as revealed by the crystal structure of a ternary complex at 1.25 Å resolution. Structure. 2000;8(10):1049–1058. doi:https://doi.org/10.1016/S0969-2126(00)00502-5.

4. Otterbein LR, Kordowska J, Witte-Hoffmann C, Wang CLA, Dominguez R. Crystal Structures of S100A6 in the Ca2+-Free and Ca2+-Bound States: The Calcium Sensor Mechanism of S100 Proteins Revealed at Atomic Resolution. Structure. 2002;10(4):557–567. doi:https://doi.org/10.1016/S0969-2126(02)00740-2.

5. Lewit-Bentley A, Réty S. EF-hand calcium-binding proteins. Current opinion in structural biology. 2000;10(6):637–643.

6. Yao N, Ledvina PS, Choudhary A, Quiocho FA. Modulation of a salt link does not affect binding of phosphate to its specific active transport receptor. Biochemistry. 1996;35(7):2079–2085.

7. Ménétrey J, Macia E, Pasqualato S, Franco M, Cherfils J. Structure of Arf6–GDP suggests a basis for guanine nucleotide exchange factors specificity. Nature structural biology. 2000;7(6):466–469.

8. Pasqualato S, Ménétrey J, Franco M, Cherfils J. The structural GDP/GTP cycle of human Arf6. EMBO reports. 2001;2(3):234–238.

9. Müller C, Schlauderer G, Reinstein J, Schulz GE. Adenylate kinase motions during catalysis: an energetic counterweight balancing substrate binding. Structure. 1996;4(2):147–156.

10. Müller CW, Schulz GE. Structure of the complex between adenylate kinase from Escherichia coli and the inhibitor Ap5A refined at 1.9 Å resolution: A model for a catalytic transition state. Journal of molecular biology. 1992;224(1):159–177.

11. Lennon BW, Williams CH, Ludwig ML. Twists in catalysis: alternating conformations of Escherichia coli thioredoxin reductase. Science. 2000;289(5482):1190–1194.

12. Waksman G, Krishna TS, Williams Jr CH, Kuriyan J. Crystal Structure of Escherichia coli Thioredoxin Reductase Refined at 2 A Resolution: Implication for a Large Conformational Change during Catalysis. J Mol Biol. 1994;.
