# Supplementary material for: Rosetta design with co-evolutionary information retains protein function
Source: PLoS Comput Biol. 2021 Jan 19;17(1):e1008568. doi: 10.1371/journal.pcbi.1008568 (PMC7815116; doi:10.1371/journal.pcbi.1008568)
Supplement: S2 Supplement — (PDF) [file pcbi.1008568.s002.pdf]

Section 2

Rosetta Design Protocols

2.1 Clean and relax

All proteins were cleaned and relaxed before design. An ensemble of five relaxed structures was used as a starting point for all designs.

```
./Rosetta/tools/protein_tools/scripts/clean_pdb.py $PDBid $CHAIN
```

Listing 2.1. PDB cleaning commands

```
./Rosetta/main/source/bin/relax.default.linuxgccrelease -s $PDB -use_input_sc -
    nstruct 5 -relax:constrain_relax_to_start_coords -scorefile relax.fasc -
    out:suffix _relax
```

Listing 2.2. Rosetta relax commands

```
./Rosetta/main/source/bin/rosetta_scripts.default.linuxgccrelease @design.
    options
-parser:protocol /design.xml -out:suffix_design -scorefile design.fasc -s
    $pdb
```

Listing 2.3. RosettaScripts design command file for unconstrained designs.

```
<ROSETTASCRIPTS>
        <SCOREFXNS>
        </SCOREFXNS>
        <TASKOPERATIONS>
                <InitializeFromCommandline name="ifcl"/>
        <ReadResfile name="rrf" filename="design.resfile"/>
        </TASKOPERATIONS>
        <MOVERS>
        <PackRotamersMover name="design" scorefxn="REF2015" task_operations="
            ifcl,rrf" />
        </MOVERS>
        <FILTERS>
        </FILTERS>
        <APPLY_TO_POSE>
        </APPLY_TO_POSE>
        <PROTOCOLS>
        <Add mover="design" />
        </PROTOCOLS>
        <OUTPUT scorefxn="REF2015" />
</ROSETTASCRIPTS>
```

Listing 2.4. RosettaScripts XML file for unconstrained designs.

2.2 Unconstraint Rosetta Single State Design (RoSSD)

The following options were used to design proteins in the benchmark without any additional constraints (control group).

```
-linmem_ig 5 -ex1 -ex2 -nstruct 10000
design.resfile:
ALLAAxc
start
```

Listing 2.5. Rosetta design options and residue file for unconstrained designs.

2.3 Design with co-evolutionary constraints (ResCue)

The following options and commands were used for the new ResCue protocol (with the same options and resfile as above).

```
./Rosetta/main/source/bin/rosetta_scripts.default.linuxgccrelease @design.
    options
-parser:protocol design.xml -out:suffix _design -scorefile design.fasc -s  $pdb
```

Listing 2.6. RosettaScripts command for co-evolutionary constraint designs.

```
<ROSETTASCRIPTS>
        <SCOREFXNS>
                <ScoreFunction name="scorefxn_cst" weights="ref2015.wts">
                        <Reweight scoretype="res_type_linking_constraint"
                            weight="1.0"/>
                </ScoreFunction>
                <ScoreFunction name="scorefxn" weights="ref2015.wts"/>
        </SCOREFXNS>
        <TASKOPERATIONS>
                <InitializeFromCommandline name="ifcl"/>
                <ReadResfile name="rrf" filename="design.resfile"/>
        </TASKOPERATIONS>
        <MOVERS>
                <AddResidueCouplingConstraint name="favor" tensor_file=".
                    tensorBinary.bin" index_file="indexList" strength="1.0"
                    alphabet="ARNDCQEGHILKMFPSTWYV-"/>
                <PackRotamersMover name="design" scorefxn="scorefxn_cst"
                    task_operations="ifcl,rrf" />
        </MOVERS>
        <FILTERS>
        </FILTERS>
        <PROTOCOLS>
                <Add mover="favor" />
                <Add mover="design" />
        </PROTOCOLS>
        <OUTPUT scorefxn="scorefxn" />
</ROSETTASCRIPTS>
```

Listing 2.7. RosettaScripts XML file for co-evolutionary constraint designs.

2.4 RECON Multistate Designs (MSD)

Following commands and options were used for RECON multistate design (with the same Options and resfile as above):

```
./Rosetta/main/source/bin/recon.default.linuxgccrelease @design.options -parser
    :protocol design.xml -out:suffix _multiDesign -scorefile design.fasc -s
    $pdb $pdb2
```

Listing 2.8. RosettaScripts design command for RECON MSD design.

```
<ROSETTASCRIPTS>

        <TASKOPERATIONS>
                <InitializeFromCommandline name="ifcl"/>
        </TASKOPERATIONS>
        <MOVERS>

                <PackRotamersMover name="design" scorefxn="REF2015"
                    task_operations="ifcl" />

                <MSDMover name="msd1" design_mover="design" constraint_weight
                    ="0.5" resfiles="design.resfile, design.resfile" />
                <MSDMover name="msd2" design_mover="design" constraint_weight
                    ="1" resfiles="design.resfile, design.resfile"/>
                <MSDMover name="msd3" design_mover="design" constraint_weight
                    ="1.5" resfiles="design.resfile, design.resfile" />
                <MSDMover name="msd4" design_mover="design" constraint_weight
                    ="2" resfiles="design.resfile, design.resfile" />

                <FindConsensusSequence name="finish" scorefxn="REF2015"
                    resfiles="design.resfile, design.resfile" />
        </MOVERS>
        <FILTERS>
        </FILTERS>
        <PROTOCOLS>
                <Add mover="msd1" />
                <Add mover="msd2" />
                <Add mover="msd3" />
                <Add mover="msd4" />

                <Add mover="finish" />
        </PROTOCOLS>
        <OUTPUT scorefxn="REF2015" />
</ROSETTASCRIPTS>
```

Listing 2.9. RosettaScripts XML file for RECON design.

2.5 Design with a position specific scoring matrix (PSSM)

Following commands were used to design with a PSSM.

```
./Rosetta/main/source/bin/rosetta_scripts.default.linuxgccrelease
@design.options -parser:protocol design.xml -out:suffix _design -scorefile
    design.fasc -s  $pdb
```

Listing 2.10. RosettaScripts design command and XML file for design constraint with PSSM.

```
<ROSETTASCRIPTS>
        <SCOREFXNS>
        <ScoreFunction name="scorefxn" weights="ref2015.wts">
                <Reweight scoretype="res_type_constraint" weight="0.0"/>
        </ScoreFunction>
        <ScoreFunction name="scorefxn_cst" weights="ref2015.wts">
                <Reweight scoretype="res_type_constraint" weight="1.0"/>
        </ScoreFunction>
        </SCOREFXNS>
        <TASKOPERATIONS>
                <InitializeFromCommandline name="ifcl"/>
                <ReadResfile name="rrf" filename="design.resfile"/>
        </TASKOPERATIONS>
        <MOVERS>
                <FavorSequenceProfile name="favorSequence" scaling="global"
                    weight="5" pssm="pssm.txt" scorefxns="scorefxn_cst" />
                <PackRotamersMover name="design" scorefxn="scorefxn_cst"
                    task_operations="ifcl,rrf" />
        </MOVERS>
        <FILTERS>
        </FILTERS>
        <PROTOCOLS>
                <Add mover="favorSequence"/>
                <Add mover="design" />
        </PROTOCOLS>
        <OUTPUT scorefxn="scorefxn" />
</ROSETTASCRIPTS>
```

Listing 2.11. RosettaScripts XML for design constraint with PSSM.

2.6 Design favoring the wild-type sequence

Following commands were used to design with a limited amount of mutations.

```
./Rosetta/main/source/bin/rosetta_scripts.default.linuxgccrelease
@design.options -parser:protocol design.xml -out:suffix _design -scorefile
    design.fasc -s $pdb -"parser:script_vars weight=WEIGHT"
```

Listing 2.12. RosettaScripts design command and XML file for design constraint to the native sequence.

Table 2.1. Weights used for the FavorNative protocol for each benchmark protein.

PDB1	PDB2	Weight	Protein Description
1CKK	1CFD	1.8	Calmodulin
1EOS	2J5X	1.4	G-protein Arf6
6Q21	4Q21	1.25	RasH
1TDE	1F6M	1.0	Thioredoxin reductase
1QUK	1OIB	1.0	Phosphate-binding protein
2LAO	1LAF	0.8	LAO Binding protein
1K9P	1K9K	0.6	S100A6
1AKE	4AKE	0.7	Adenylate kinase
1HKA	1Q0N	0.7	HPPK
1D5W	1DBW	0.4	FixJ

```
<ROSETTASCRIPTS>
    <SCOREFXNS>
        <ScoreFunction name="scorefxn" weights="ref2015.wts">
            <Reweight scoretype="res_type_constraint" weight="0.0"/>
        </ScoreFunction>
        <ScoreFunction name="scorefxn_cst" weights="ref2015.wts">
            <Reweight scoretype="res_type_constraint" weight="%%weight%%"/>
        </ScoreFunction>
    </SCOREFXNS>
    <TASKOPERATIONS>
        <InitializeFromCommandline name="ifcl"/>
            <ReadResfile name="rrf" filename="/home/erteltm/moritz/
                Protein_Designs/Constrained/Full/design.resfile"/>
    </TASKOPERATIONS>
    <MOVERS>
        <FavorSequenceProfile name="favorSequence"  weight="1.3" use_current="
            true" matrix="IDENTITY" scorefxns="scorefxn_cst" />
        <PackRotamersMover name="design" scorefxn="scorefxn_cst"
            task_operations="ifcl,rrf" />
    </MOVERS>
    <FILTERS>
    </FILTERS>
    <APPLY_TO_POSE>
    </APPLY_TO_POSE>
    <PROTOCOLS>
        <Add mover="favorSequence"/>
        <Add mover="design" />
    </PROTOCOLS>
    <OUTPUT scorefxn="scorefxn" />
</ROSETTASCRIPTS>
```

Listing 2.13. RosettaScripts XML for design constraint to the native sequence.

For each benchmark protein, the weight of the score function term *res_type_constraint* was optimized to roughly reflect the average native sequence recovery of the ResCue protocol. This allows to compare the coupling recovery across the protocols. Table 1 lists the used weights for the FavorNative protocol.
