# Supplementary material for: Rosetta design with co-evolutionary information retains protein function
Source: PLoS Comput Biol. 2021 Jan 19;17(1):e1008568. doi: 10.1371/journal.pcbi.1008568 (PMC7815116; doi:10.1371/journal.pcbi.1008568)
Supplement: S3 Supplement — (PDF) [file pcbi.1008568.s003.pdf]

Section 3

ResCue full length sequence logos

Here, full length weblogos are provided for the ResCue design on the benchmark dataset (Figures S1-S10). The weblogos visualize high wild-type sequence recovery over the full protein length.

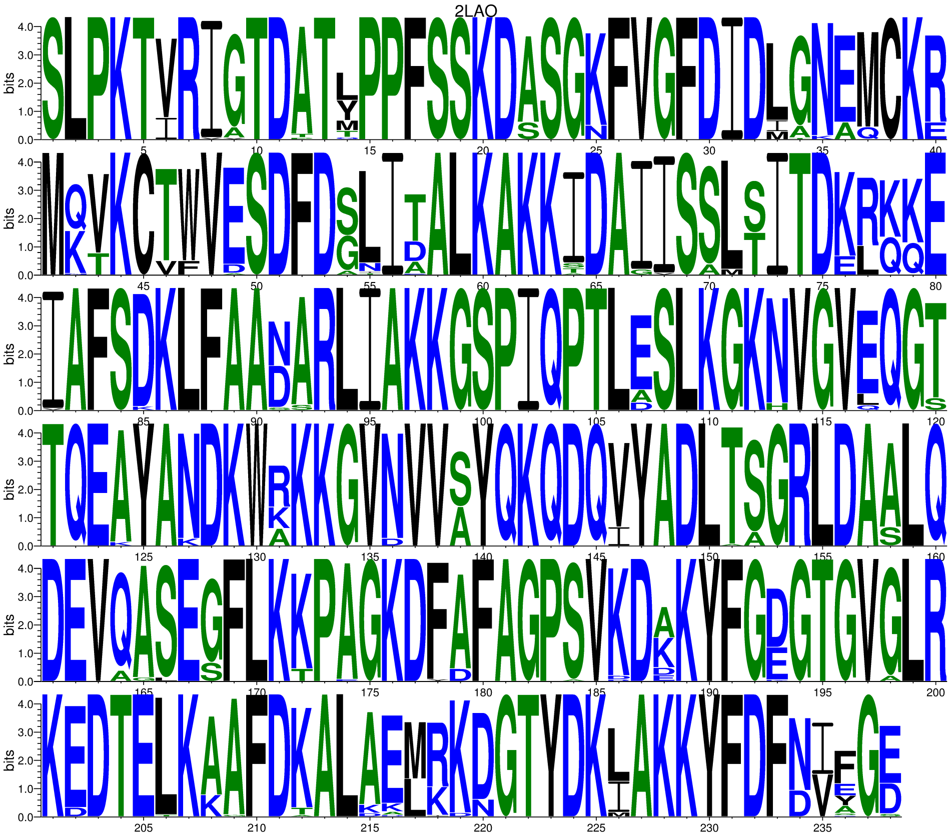

Fig 3.1. Full sequence weblogo for the ResCue design on LAO

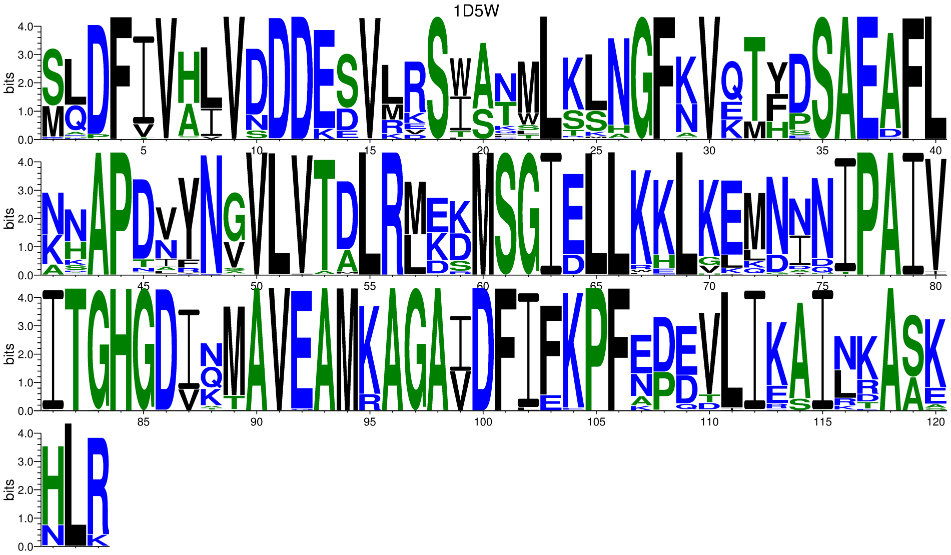

Fig 3.2. Full sequence weblogo for the ResCue design on FixJ

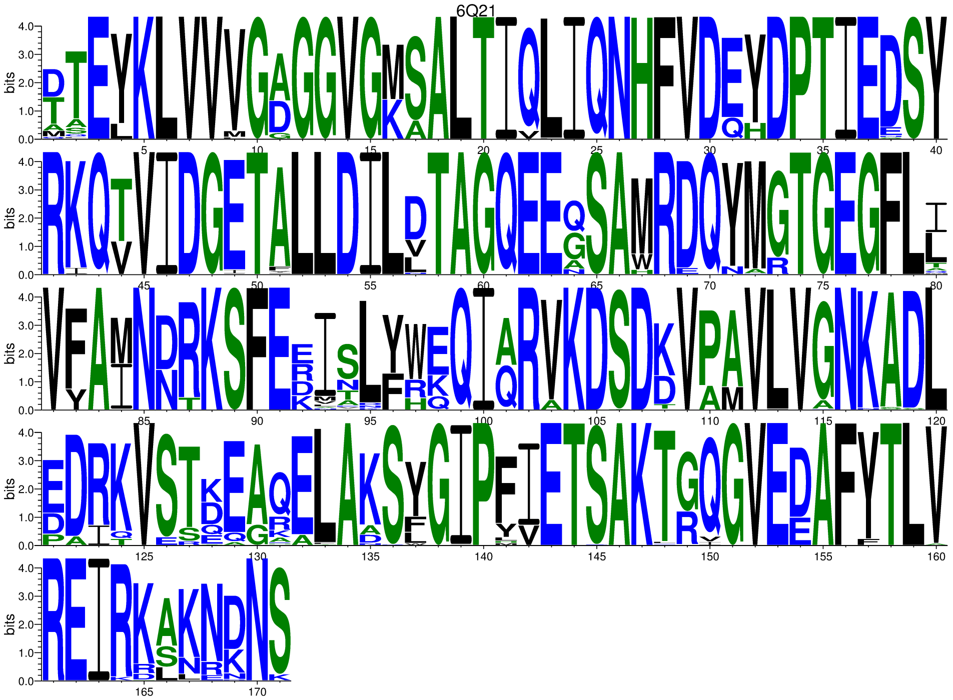

Fig 3.3. Full sequence weblogo for the ResCue design on RasH

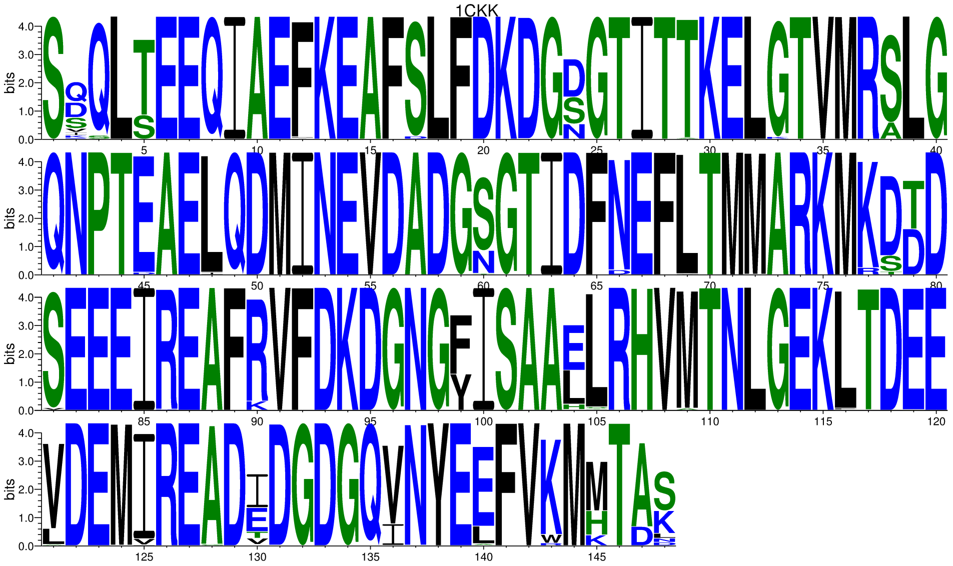

Fig 3.4. Full sequence weblogo for the ResCue design on Calmodulin

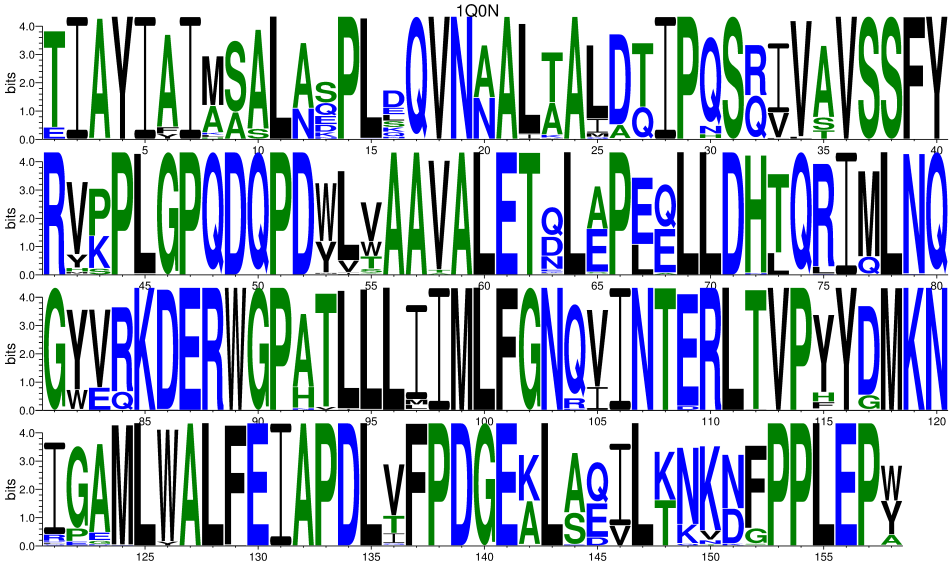

Fig 3.5. Full sequence weblogo for the ResCue design on HPPK

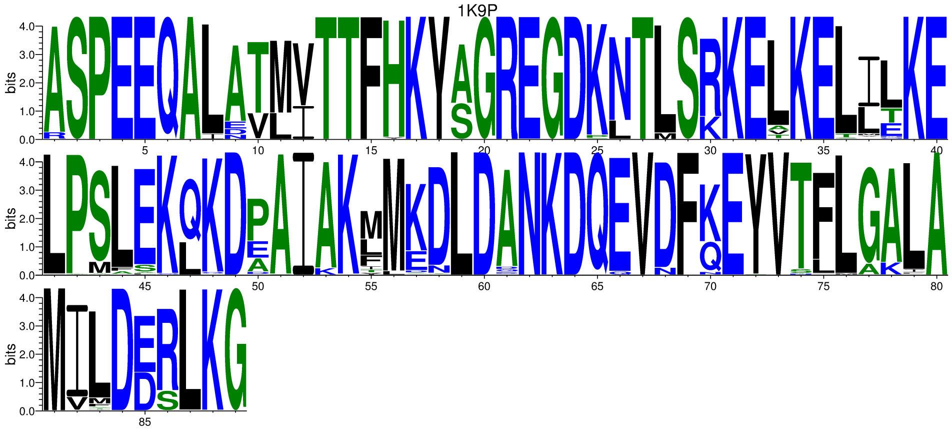

Fig 3.6. Full sequence weblogo for the ResCue design on S100A6

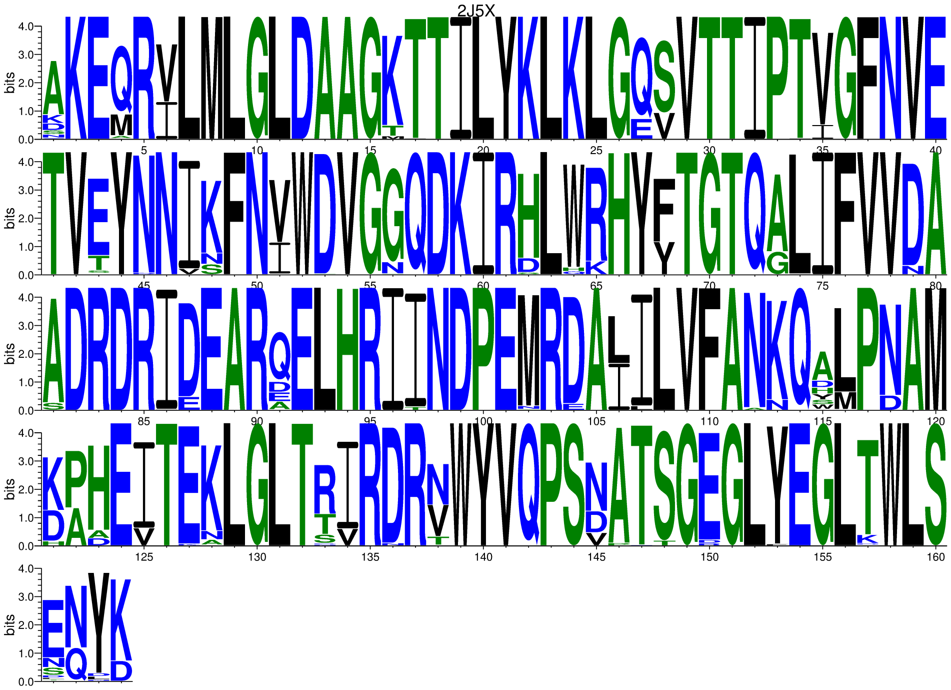

Fig 3.7. Full sequence weblogo for the ResCue design on Arf 6

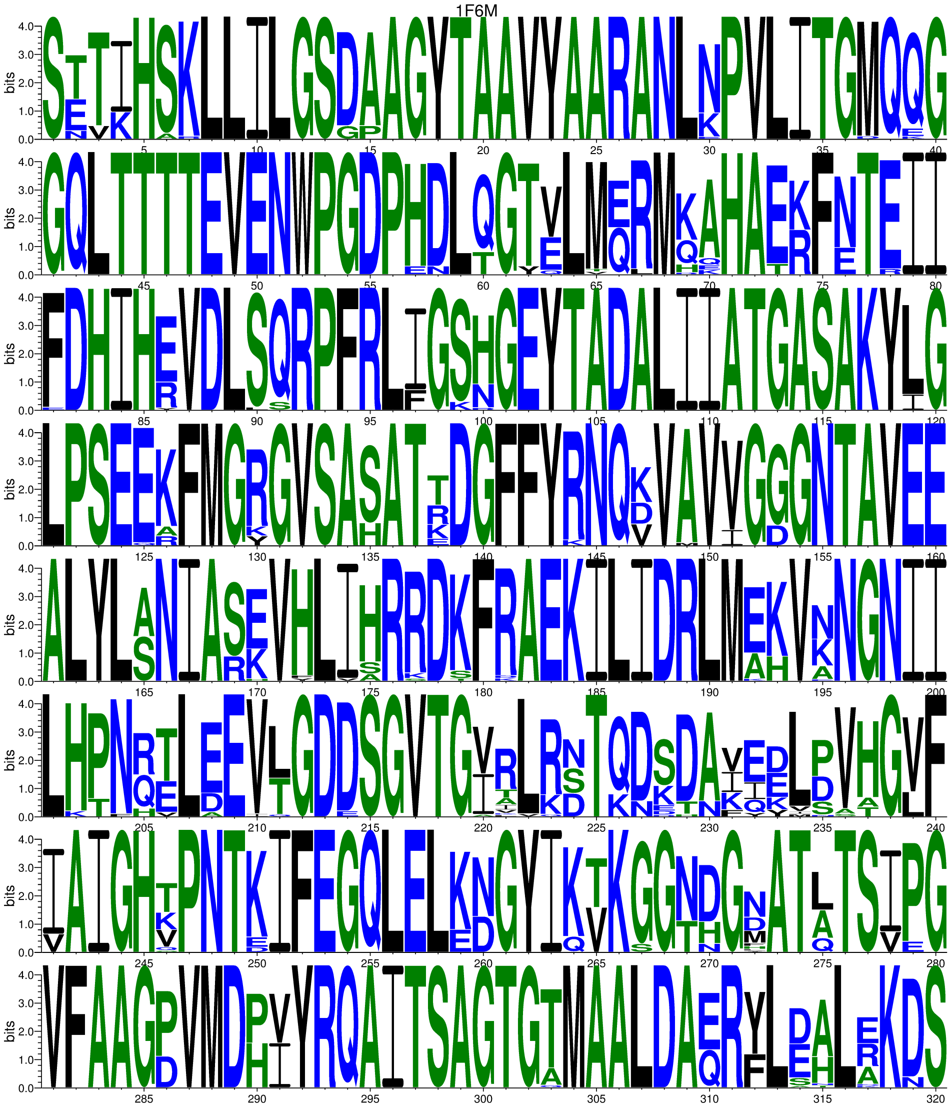

Fig 3.8. Full sequence weblogo for the ResCue design on thioredoxin reductase

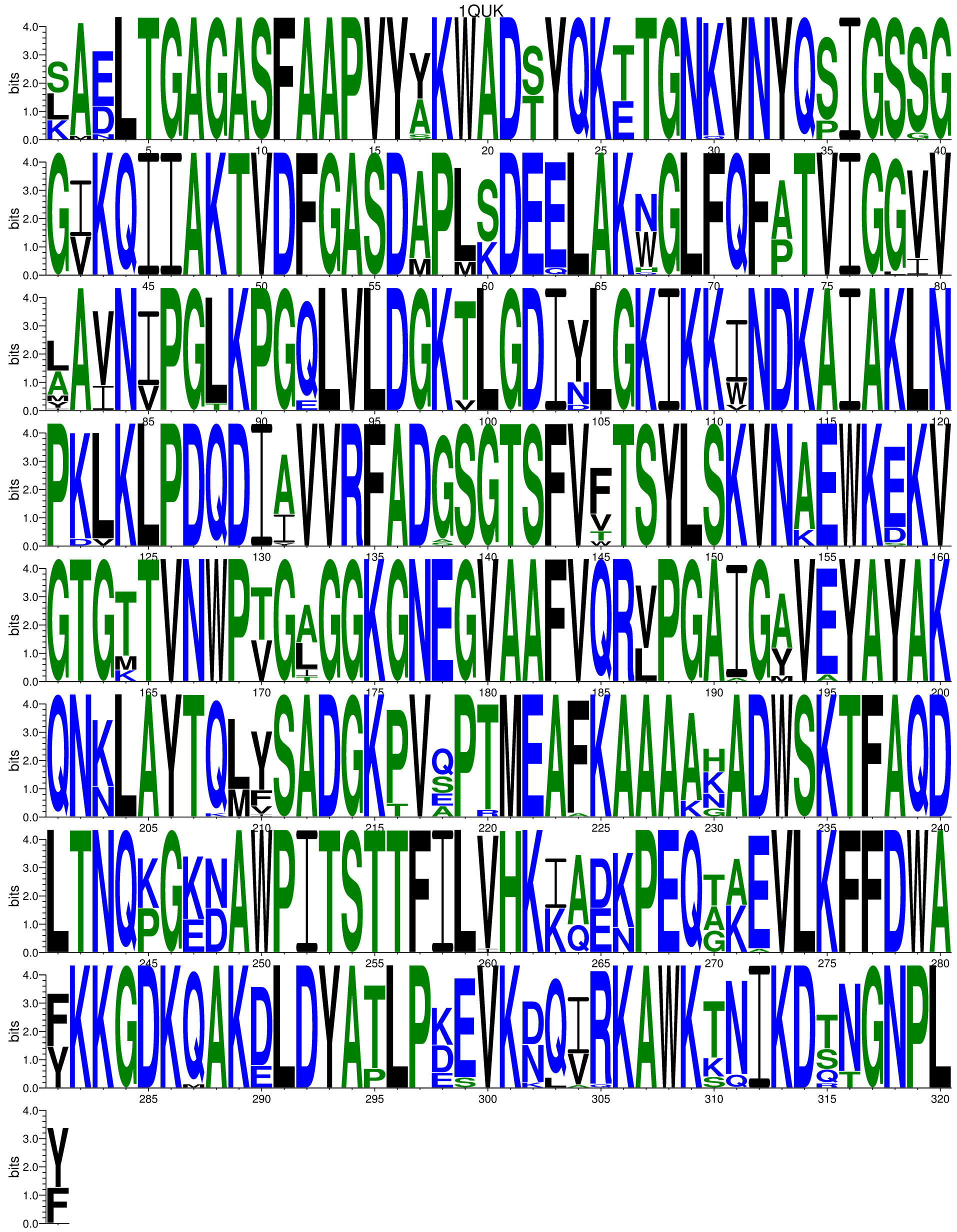

Fig 3.9. Full sequence weblogo for the ResCue design on Phosphate binding protein

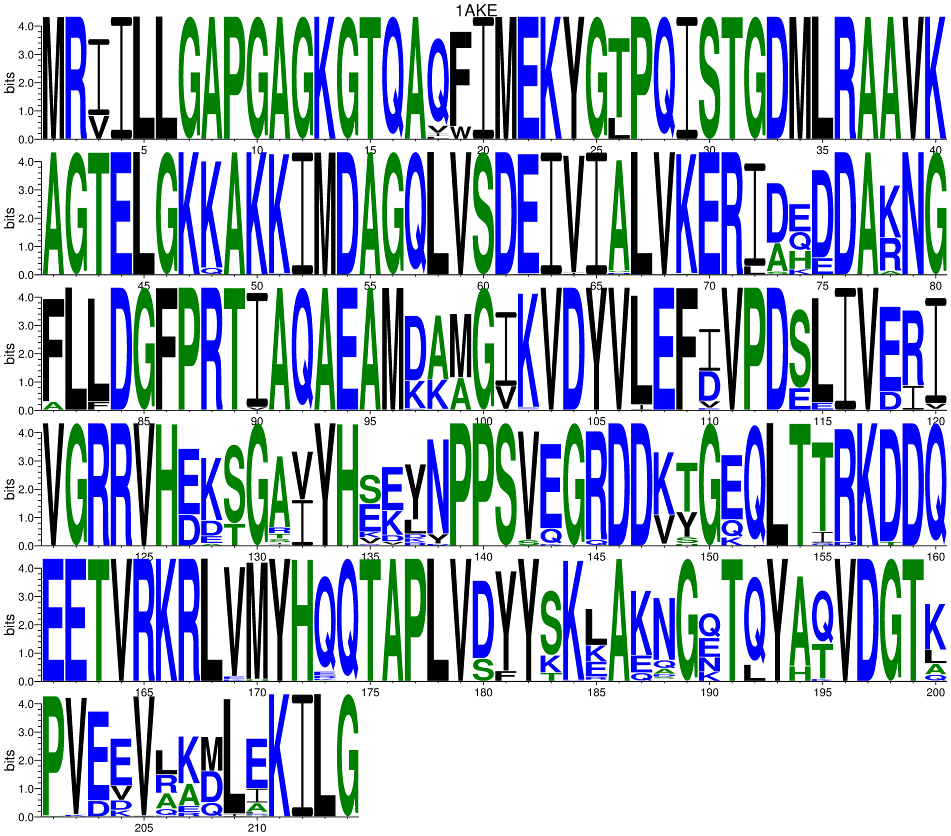

Fig 3.10. Full sequence weblogo for the ResCue design on adenylate kinase
