# Supplementary material for: Rosetta design with co-evolutionary information retains protein function
Source: PLoS Comput Biol. 2021 Jan 19;17(1):e1008568. doi: 10.1371/journal.pcbi.1008568 (PMC7815116; doi:10.1371/journal.pcbi.1008568)
Supplement: S4 Supplement — (PDF) [file pcbi.1008568.s004.pdf]

Section 4

Coupling strength of functionally relevant residues

Figure S1 shows the coupling strength cs(seq) of functional relevant residues in the benchmark proteins. Residues were chosen according when mentioned in literature as functional (see Methods of manuscript). The coupling strength cs(seq) of the chosen set of residues for each protein was calculated and visualized as a bar plot. Compared to the native sequence, the coupling strength of ResCue was on average 103±50%, followed by FavorNative with 35±55%, SeqProf with 23±58%, RECON with 20±63% and RoSSD with 4±56% (Fig8). The improvement of ResCue was statistically significant compared to all other design methods (MW p ¡ 5.0e-04) (Fig 8). The increased sequence recovery of the ResCue protocol can therefore be attributed to the collective interaction of couplings.

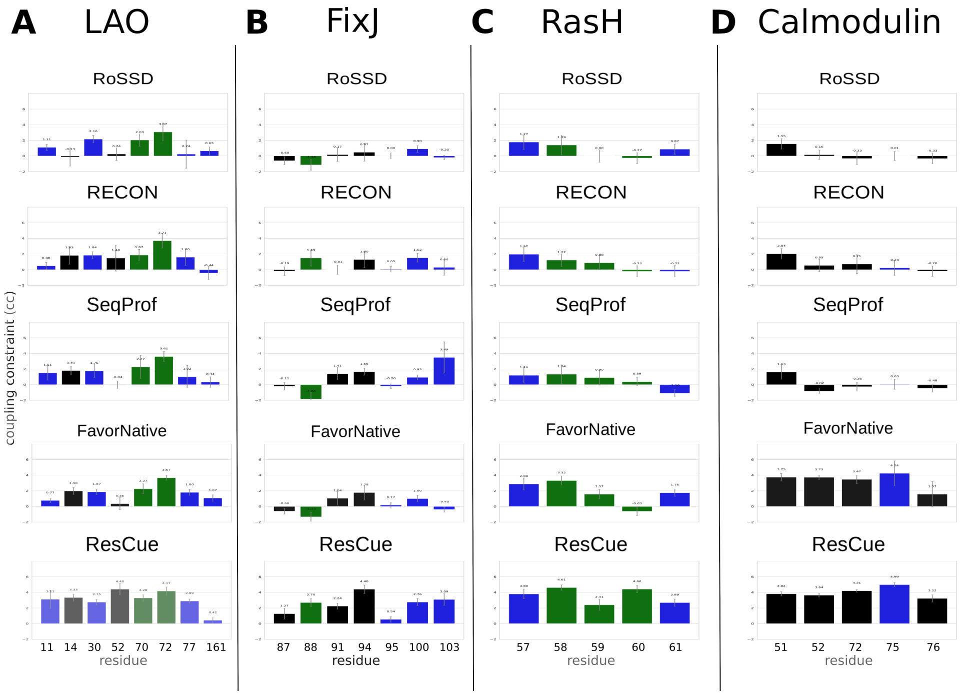

Fig 4.1. Coupling strengths for residues relevant to function. The methods are sorted by the average coupling strength, and increases in the order: RoSSD, RECON, SeqProf, FavorNative and ResCue (top to down). The observation was made for all proteins, (A) LAO binding site, eight residues. (B) FixJ dimer interface, seven residues (C) RasH binding site, five residues. (D) calmodulin-binding site, five residues.
