# Supplementary material for: Rosetta design with co-evolutionary information retains protein function
Source: PLoS Comput Biol. 2021 Jan 19;17(1):e1008568. doi: 10.1371/journal.pcbi.1008568 (PMC7815116; doi:10.1371/journal.pcbi.1008568)
Supplement: Attachment — Submitted filename: Comments_to_the_Authors_rev1.pdf [file pcbi.1008568.s005.pdf]

We would like to thank the reviewers for their constructive criticism. We updated the manuscript to address their concerns. To facilitate re-review we include all changes applied to the main text, figures, or tables in this point-by-point response letter.

1 - Specifically, the sequence recovery and similarity metrics reward proximity to the native sequence, which can be trivially achieved by simply copying the starting sequence. This copy-native design algorithm would be superior to fixed-backbone design and the other 2 algorithms in all four of the metrics shown in Figure 3 (native sequence recovery, native sequence similarity, native cluster recovery, coupling recovery) and superior to ResCue in three of the four (with a pretty similar score of 100% for the fourth, coupling recovery). It's hard to say how copy-native would perform in the rosetta energy test-- presumably it will be worse unless there is a sampling problem identifying the native sequence. A more interesting and useful test would be to do a comparison with a simple variant of the default, fixed-backbone method ('RoSSD') with a single change: addition of a favor-native sequence bonus. The weight on the favor-native bonus could be tuned to give the same total sequence recovery as for ResCue, and then the two really interesting comparisons would be (1) the rosetta energy test (Fig. 2), and (2) the coupling recovery score. Presumably ResCue will give better coupling recovery (since it is one of the energy terms being directly optimized in ResCue designs), but I wonder how much better? And what will the Rosetta energy comparison look like?

Apparently, the objective of the individual benchmarks was made insufficiently clear in the original manuscript. For example, a ‘copy-native design algorithm’ would fail the coupling recovery test. It would also defeat the purpose of designing proteins with new sequences but retained function. Thus, we clarified throughout the manuscript our choices for design algorithms and success metrics:

In the Introduction “Pairwise sequence requirements in natural proteins are a consequence of maintaining thermodynamic stability, structural flexibility (plasticity), and other requirements for protein function, such as recognizing interaction partners, catalyzing chemical reactions, and many more. Computational protein design with Rosetta primarily favors thermodynamic stability and is conceptually unaware of couplings required for protein flexibility and/or function. The premise of this study is that this restriction in evolutionary tolerated sequence space is not reflected in Rosetta designed proteins. This leads to design solutions that are thermodynamically stable but might change flexibility or lose function. While custom protocols for a specific design task can circumvent this shortcoming, we wondered about a general approach to maintain native-like couplings in the sequences designed beyond the couplings dictated by thermodynamic stability. For this study, we evaluate a number of computational design protocols in Rosetta: 1) One biased towards the wild-type sequence as a baseline for comparison, 2) Design with a sequence profile, which encodes the sequence space as observed in functional proteins, 3) RECON multi state design, which has the potential to capture couplings critical for protein plasticity, and 4) Constraining co-evolving residues directly in the Rosetta design process.“

In the Introduction: “We use native sequence recovery as one of our metrics of design success in order to facilitate comparison with other studies. Although it might appear counter-intuitive to use this measure to assess coupling recovery, we argue that it is a useful metric as increased coupling recovery will imply increased sequence recovery. Our method achieves high recovery rates by conserving networks of co-evolving residue pairs, in contrast to an alternative approach that trivially increases

sequence recovery rates by limiting the number of mutations. We show, that our method is superior in recapitulating the wild-type residues especially in functionally active sites compared to other approaches and thus is suitable to retain function during design.

The following updated figures include results of a design that limits the number of mutations.

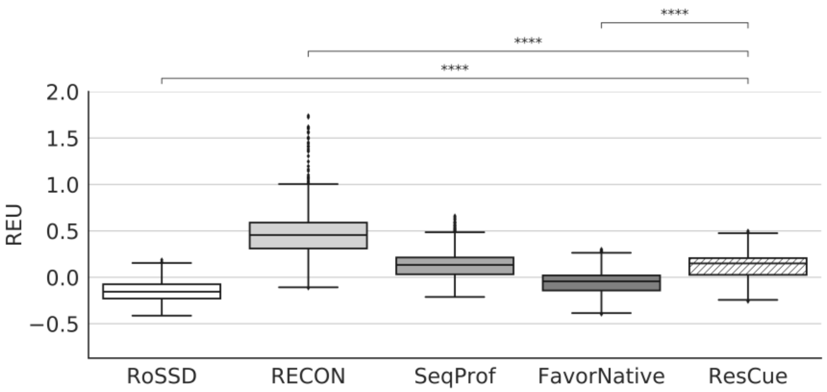

Fig 2. **Distribution of Rosetta total energies for the full benchmark design.** Energies are given in Rosetta energy units (REU) relative to the wild-type on a per residue basis for the five design protocols. For this figure and all subsequent boxplots, the median is indicated as a black line; boxes depict the interquartile range (IQR), whiskers represent 1.5 x IQR. Results of a two-sided Mann-Whitney-Wilcoxon test are indicated as follows: * ≙ 1.00e-02 < p <= 5.00e-02, * ≙ 1.00e-03 < p <= 1.00e-02, * ≙ 1.00e-04 < p <= 1.00e-03, *** ≙ p <= 1.00e-04.

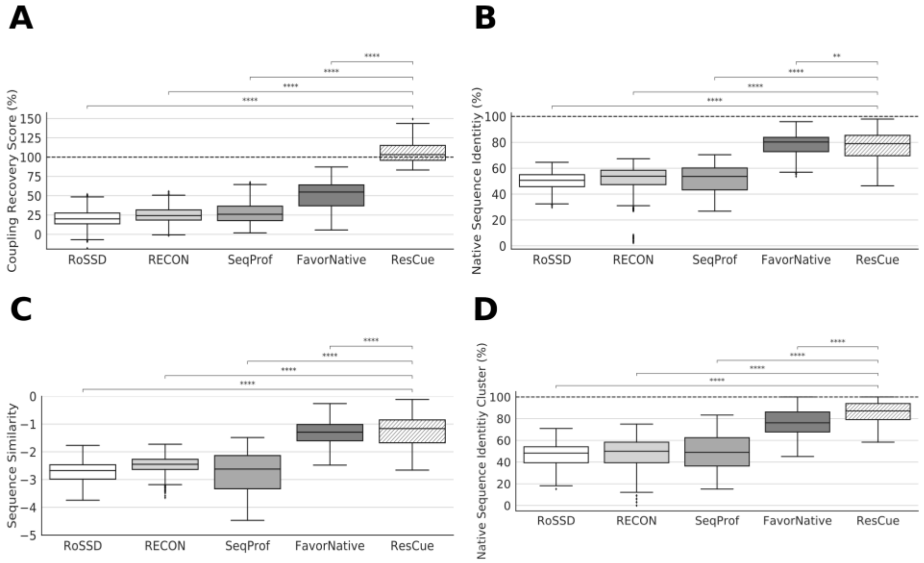

Fig 1. **Performance of four different design approaches.** (**A**) Coupling recovery scores *crs* deduced for all designed sequences. The 100 % value is indicated by the dashed line. (**B**) Native sequence recovery *nsr* of the four design approaches. (**C**) Sequence similarity *seqsim* of the full-length sequences. (**D**) Native sequence recovery nsr_{CN} of residues found in clustered networks. The scores are summarized with boxplots as explained above. Results of a two-sided Mann-Whitney-Wilcoxon test are indicated as follows: * ≙ 1.00e-02 < p <= 5.00e-02, * ≙ 1.00e-03 < p <= 1.00e-02, * ≙ 1.00e-04 < p <= 1.00e-03, *** ≙ p <= 1.00e-04).

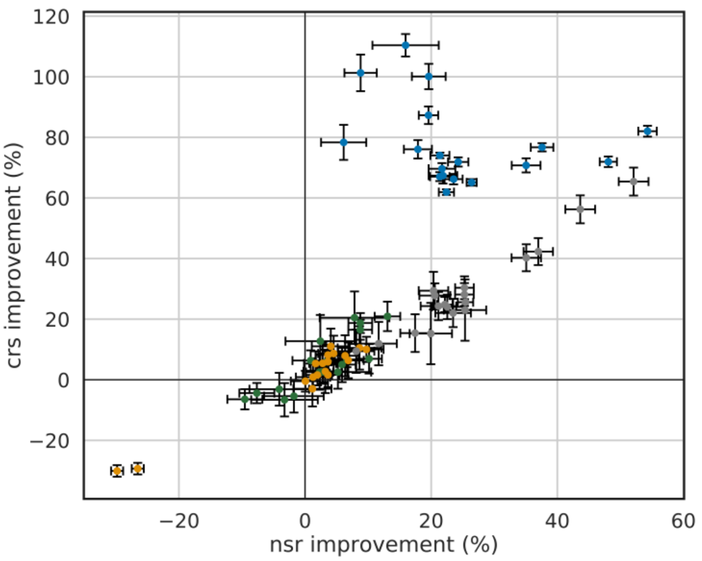

Fig 3. **Improvement of native sequence recovery values and coupling recovery scores for the two conformational states of all ten benchmark proteins.** For each state, the *nsr* value of the default Rosetta protocol was subtracted from the *nsr* value reached by one of the other protocols. The *crs* values were processed analogously. The following color code indicates the design protocols: RECON (orange), SeqProf (green), ResCue (blue), and FavorNative (gray).

In the Results: "The FavorNative design energies remain on average close to the wild-type energies (-0.057 REU). Most likely, for several positions residue choices with similar energies exist and FavorNative helps to select the native residue. As expected, sequence recovery increases substantially with the FavorNative method, which is a trivial result as the correct solution is input into the method. Thus, this approach would not allow the design of new sequences that retain structure, plasticity, and function. Details on the execution of each experiment and the used constraint weights can be found in Supplement Section 2."

2 - I am also curious to understand how the cs(seq) term compares with the total log-likelihood of seq in the GREMLIN graphical model. It seems like they are similar, except maybe that cs(seq) only has the pairwise terms, not the one-body (single-position) terms that are also present in the log-likelihood. This makes me wonder how ResCue would compare to designing with the full GREMLIN (pseudo)log-likelihood as the additional energy term, rather than the pairwise interactions...

While we agree with the reviewer that using the (pseudo)log-likelihood might be an interesting experiment in a different context, it does not align with the hypothesis and approach of the study. We clarify this point now in the manuscript:

In the Methods: "The coupling strengths $DC_{i,j}(aa_x, aa_y)$ from the Markov Random Field (MRF) tensor were used to restrain designs with ResCue. MRF-values were preferred over the derived GREMLIN (pseudo)log-likelihood values [24] for two reasons: Firstly, (pseudo)log-likelihood values combine coupling strength and amino acid preferences at a certain position. In the MRF tensor, both values are listed separately. Here, we wanted to focus on coupling strengths. If desired, the user can utilize the already m shed FavorSequenceProfile term in addition to the ResCue coupling weight to incorporate a sequence conservation term (present in the log-likelihood). Secondly, usage of coupling strengths allows favoring correlations and penalizing anti-correlations. In contrast, the (pseudo)log-likelihood values reflect absolute coupling strengths and thus ignores this information."

3 - It wasn't completely clear which of the two structures was used in each benchmark case.

We clarified in the Methods: "For all single state design protocols, both structures were used as starting points and the results were pooled."

4 - "Note, that the crs value can be larger than 100% which would suggest that the designed sequences fulfill additionally constraints not found in the native sequences, but in homologs" -- it's also worth pointing out here that the crs is directly being optimized as part of the energy function during sequence optimization, so the fact that it improves relative to the native is not so surprising. More interesting would be to evaluate coupling recovery in some way that isn't directly part of the energy function.

Coupling recovery without bias in the scoring function was demonstrated with methods that may support couplings indirectly (RECON and SeqProf), as discussed in the paper. We would like to refer the reviewer to the sections that were revised in response to comment 1.

5 - " in spacial proximity "

We corrected the typo.

6 - "and a seqsim value of 2.72" should be -2.72; also it would be helpful if "seqsim" were defined where first used...

We thank the reviewer for pointing this out and corrected the sign.

Sequence similarity (seqsim) is introduced in before its first usage.

7 - "Rash H," should be "Ras H"

All occurrences of Rash H were corrected

8 - "Analyzing how different design approaches sample five exemplary residues" -- how were these 5 residues chosen?? Where subsets of positions have to be chosen for visual evaluation, perhaps full sequence logos could be provided in the supplementary material to reassure readers that the authors aren't cherry-picking.

The choice of the residues for all benchmark proteins (mined from literature) is now mentioned **in the Results**: "As expected, these residues were often described as functionally relevant in the literature (see discussion for individual proteins below)."

We elaborate for the special case of calmodulin **in the Results**: "The set of functionally relevant residues consists of 25 (34 - 53, 72 - 76) and the network is located at the N-terminal hydrophobic pocket anchoring T444 of the CaMKK peptide (Fig 5D, Fig 6D). Due to the large number of residues reported as functionally relevant, five exemplary residues were chosen with approximately similar distance: M51, I52, M72, K75, M76. The five residues are part of the coupling network and are crucial for the CaMKK binding. The results highlight how our co-evolutionary approach biases the design towards the native amino acids (Fig 4D)."

Full sequence logos were added in supplementary section 3, Figure 7 was updated to include the new experiment.

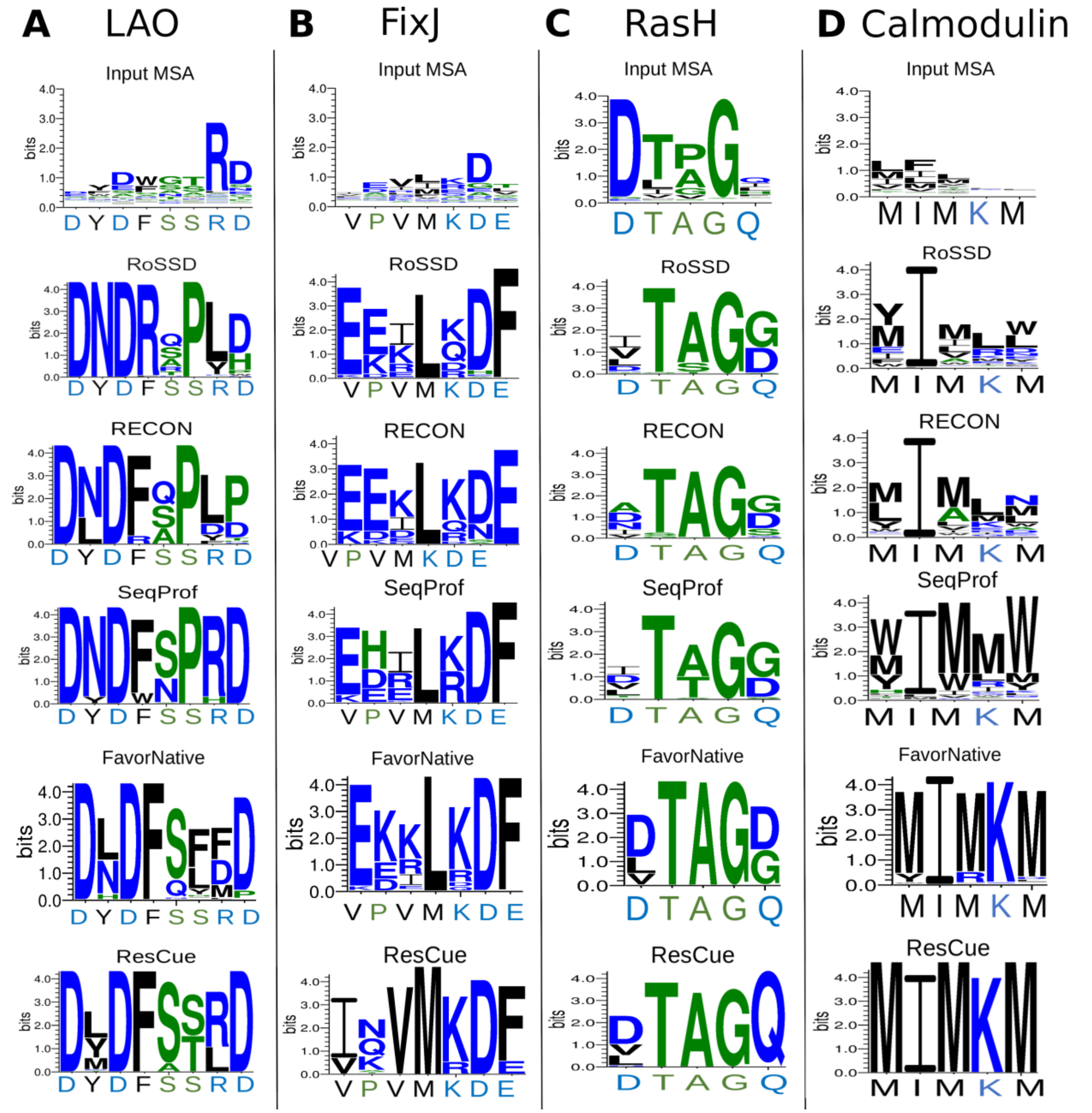

Fig 4. **Sequence logos resulting from five design protocols.** The native sequences are listed below the logos. (**A**) LAO binding site, eight residues. (**B)** FixJ dimer interface, seven residues (**C**) RasH binding site, five residues. (**D)** calmodulin-binding site, five residues.

Reviewer #2:

1 - First, the authors do not address the tradeoff between recovering couplings and optimizing their physical energy function in a series way. They write: "As noted above, it is essential, to balance carefully between having an efficient coupling restraint and designing physically realistic sequences as dictated by the Rosetta energy function." However, as far as I can tell they don't describe at all how they achieved this balance, or how we would know that the balance is good. Fig 2 shows that their method achieves better Rosetta total energies compared with the RECON and SeqProf methods, but their energies are still significantly higher than the RoSSD method (naive design without bias). How are we supposed to judge whether this difference in score is a significant cost? What efforts were made to balance their bias with the physical elements in the score function? One potential way to look at this would be to show what the total energy scores of the native proteins are before design, to show how much improvement is lost by enforcing the couplings.

We now present the REU values relative to the Rosetta energy of the input structure. A brief discussion the guide the interpretation of REU values were added **in the Results**:

“By restraining Rosetta to bias the sampling towards a desired goal, the energy landscape is modified. As a result, when reevaluating the solutions with the unmodified energy function, the energy can and often does increase (get worse). The ResCue protocol (Supplement Section 2.3) was parametrized to produce designs with Rosetta energies comparable to the established SeqProf method and substantially increased coupling recovery. In order to assess the energy increase of the different design approaches, we determined the difference of the Rosetta total energy to the relaxed wild-type structure with the best energy, normalized by protein length. As expected, all design approaches with constraints had significantly higher Rosetta energies compared to the relaxed wild type (Fig 2), (Mann–Whitney U test (MW) $p < 1.0e-04$ for all three comparisons).”

In this context, please also review our response to comment 1 of reviewer 1.

And **in the Discussion**:

“The high native sequence recovery of ResCue, the fact that native sequences are close to optimal to their structure [14], and the experimental evidence that coupling guided design can generate novel sequences with stability similar to the wild type [42-44] suggests that the slightly increased Rosetta energy values is thermodynamically acceptable.”

2 - Second, it is unclear how exactly this method would be used. Throughout the paper, native sequence recovery is referred to as a positive metric, and the authors write that increased nsr is a goal of their method: "Our ResCue design showed a significant increase both in the nsr and the seqsim 183 values, which were 70% and -1.20, respectively. Compared to the other design

184 approaches, these improvements were statistically significant (MW p < 5.0e-04 for nsr 185 and seqsim).". But of course it is trivial to get 100% native sequence recovery by avoiding design and outputting the starting sequence. So native sequence recovery can't be the only goal of the work. Could the authors be specific about what they are trying to optimize and how their method helps?

First, we would like to refer the reviewer to the answer we gave on reviewer 1 first comment. Second, we summarized the utility of the method **in the Discussion**:

“Primary motivation of ResCue is to demonstrate, that co-evolutionary information can be leveraged for a Rosetta design algorithm that yields more natural sequences while conserving couplings that, at least in some cases, will be critical for plasticity or function, i.e. properties of the protein that are ‘hidden’ in a single structure/sequence pair. These couplings would encode required properties of the sequence but cannot be derived from a single conformation and in the absence of all binding partners. The Rosetta scoring function will optimize thermodynamic stability [6] of a single conformation but miss other aspects of plasticity or function. The altered scoring method with ResCue can be exploited 1) to explore a sequence space more similar to native sequences, 2) for conservative re-engineering while keeping known and unknown functions intact, or similar 3) to design on one structural conformation while not destabilizing other possible conformations. Alternative design protocols that can indirectly inform the design process about pairwise residue couplings were discussed: These are firstly, the design on multiple conformations (RECON), and secondly, design with a sequence profile. Amongst these, ResCue generates sequences most similar to the wild type and conserves the most plausible functionally important residues and couplings. Nonetheless, future experimental studies are required to confirm the suitability of our approach for the different suggested design scenarios.”

Two small comments to improve the paper:

3- -For benchmark proteins, could the paragraph "To study the coupling networks in detail, we present results for four benchmark proteins, LAO, FixJ, RasH, and calmodulin, which we have chosen for the following reasons:" go in the same odrer as the actual following information? Calmodulin is described first in the summary paragraph but last in the text.

The issue was corrected **in the Results**:

“First, the binding site residues of LAO play an essential role in stabilizing both the closed and the open state. Second, FixJ residues are involved in dimerization. Third, RasH uses two highly flexible switch domains to bind GTP. Fourth, residues crucial for peptide binding in calmodulin are only moderately conserved, which complicates efforts to recognize and recapitulate them.”

4 - -For Fig 7, please show the weblogo from the natural MSA as well as for the designed sequences. Also, these weblogos don't really help illustrate coupling. In addition to the weblogos, it would be good to show NxN matrices (where N is the number of positions) illustrating the level of mutual information (or some other metric?) between each of the positions in the weblogos. This would clearly show that coupling is present in the natural MSA, and whether it is present or absent in the four different methods.

Weblogos of the MSA was added to Figure 7 and in Supplement Section 3. Supplement Section 4 was added which shows the position-specific coupling strength in a bar plot. Methodological description **in the Results**:

"The coupling strength cs(seq) of the chosen set of residues for each protein was calculated and visualized as a bar plot. Compared to the native sequence, the coupling strength of ResCue was on average 103±50%, followed by FavorNative with 35±55%, SeqProf with 23±58%, RECON with 20±63% and RoSSD with 4±56% (Figure, Supplement Section 4). The improvement of ResCue was statistically significant compared to all other design methods (MW $p < 5.0e-04$) The increased sequence recovery of the ResCue protocol can therefore be attributed to the collective interaction of couplings. Full-length sequence logos of the ResCue design are provided in Supplement Section 3."

Reviewer #3:

1 - The exact intended use case for this method is a bit unclear. Is the method intended to improve protein thermostability, but for dynamic proteins? The manuscript can be strengthened by emphasizing the practical utility of the method more explicitly.

We would like to refer the reviewer to comment 2 of reviewer 2.

2 - If improving thermostability is the goal, ResCue is making the rosetta energy worse, which indicates the protein is being destabilized. This major caveat wasn't sufficiently addressed, and as the authors themselves note in the final sentence of the main text: "experimental studies are required to finally confirm the suitability of our approach."

Please see comment 1, the main goal can roughly be summarized as design with a more natural sequence space while simultaneously retaining both known and unknown function.

A similar comment regarding the interpretation of the Rosetta energy was addressed in reviewer 2 first comment above.

Experimental validation is out of scope of this study. The scientific body about co-evolutionary couplings, benchmark results with reasonable Rosetta energies, suggest stable proteins. **In the Discussion**:

"The high native sequence recovery of ResCue, the fact that native sequences are close to optimal to their structure [14], and the experimental evidence that coupling guided design can generate novel sequences with stability similar to the wild type [42-44] suggests that the slightly increased Rosetta energy is thermodynamically acceptable."

Please also review the high sequence recovery rates of functionally relevant residues (updated Figure 4, reviewer 1)

2) Also, if stabilizing the protein is the goal, it is unclear why preserving evolutionary couplings is so essential. Since the ResCue method requires a high resolution crystal structure of the target protein, functional residues are presumably known and do not need to be inferred from

a MSA. The current state-of-the-art method for protein stabilization using MSAs and Rosetta is PROSS from Sarel Fleishman's group. While PROSS is not aware of evolutionary coupling, it has been demonstrated with experimental methods to stabilize proteins while preserving their function. Would it be fair to compare ResCue to PROSS?

The choice of high-resolution structures for the benchmark was explained more clearly **in the Methods**:

"To not disadvantage other non-ResCue Rosetta design protocols with low quality protein structures, only structures with an experimental resolution of 3Å or better were accepted."

PROSS was mentioned **in the Discussion**:

"It was previously shown that selecting a small number of mutations based on conservation information in sequence alignments can improve expression rates and predict improved protein stability [15]. This approach however failed to allow mutations in proximity of binding partners and co-factors to prevent activity loss in the first place. By leveraging co-evolutionary information, ResCue is going beyond the task of thermodynamic stabilization and can be exploited to re-design proteins including its functionally relevant sites, even when properties crucial for function are not encoded in one sequence/structure pair."
